# A phase I trial to evaluate the safety and pharmacokinetics of low-dose methotrexate as an anti-malarial drug in Kenyan adult healthy volunteers

**DOI:** 10.1186/1475-2875-10-63

**Published:** 2011-03-16

**Authors:** Roma Chilengi, Rashid Juma, Ahmed M Abdallah, Mahfudh Bashraheil, Hudson Lodenyo, Priscilla Nyakundi, Evelyn Anabwani, Amina Salim, Gabriel Mwambingu, Ednah Wenwa, Julie Jemutai, Chemtai Kipkeu, George O Oyoo, Simon N Muchohi, Gilbert Kokwaro, Tim Niehues, Trudie Lang, Alexis Nzila

**Affiliations:** 1 Kenya Medical Research Institute (KEMRI)-Wellcome Trust Research Programme, PO Box 230, 80108 Kilifi, Kenya; 2 KEMRI-Centre for Clinical Research Nairobi, Kenya; 3 Department of Clinical Medicine and Therapeutics, University of Nairobi, Kenyatta National Hospital, P O Box 19701, 00202 KNH, Nairobi; 4 Consortium of National Health Research, Nairobi, Kenya, PO Box 29832-00202, KNH, Nairobi, Kenya; 5 Helios Klinikum Krefeld Academic Hospital, Lutherplatz 40, 47805 Krefeld, Germany; 6 Centre for Tropical Medicine, Nuffield Department of Medicine, University of Oxford, UK; 7 Departments of Chemistry and Clinical Pharmacology University of Cape Town Rondebosch 7701, Cape Town, South Africa

## Abstract

**Background:**

Previous investigations indicate that methotrexate, an old anticancer drug, could be used at low doses to treat malaria. A phase I evaluation was conducted to assess the safety and pharmacokinetic profile of this drug in healthy adult male Kenyan volunteers.

**Methods:**

Twenty five healthy adult volunteers were recruited and admitted to receive a 5 mg dose of methotrexate/day/5 days. Pharmacokinetics blood sampling was carried out at 2, 4, 6, 12 and 24 hours following each dose. Nausea, vomiting, oral ulcers and other adverse events were solicited during follow up of 42 days.

**Results:**

The mean age of participants was 23.9 ± 3.3 years. Adherence to protocol was 100%. No grade 3 solicited adverse events were observed. However, one case of transiently elevated liver enzymes, and one serious adverse event (not related to the product) were reported. The maximum concentration (C_max_) was 160-200 nM and after 6 hours, the effective concentration (C_eff_) was <150 nM.

**Conclusion:**

Low-dose methotraxate had an acceptable safety profile. However, methotrexate blood levels did not reach the desirable C_eff _of 250-400-nM required to clear malaria infection *in vivo*. Further dose finding and safety studies are necessary to confirm suitability of this drug as an anti-malarial agent.

## Background

Chemotherapy remains one of the most important tools for the management and control of malaria. The World Health Organization has recommended the use of artemisinin combination therapy (ACTs) as first-line treatments of uncomplicated malaria; ACT is also used for the treatment of malaria in pregnancy during the second and third trimesters [1]. Artemether-lumefantrine is the first ACT to be introduced in Africa [1]. Pyronaridine/artesunate and pipepraquine/dihydroartemesin are in the late development stages and have reached Phase III/IV clinical evaluation [2]. The artemisinin derivative artesunate has been recommended as a drug choice for the treatment of severe malaria, in replacement quinine [3]. However, the emergence of *Plasmodium falciparum *parasite resistant to artemisinin derivatives has been reported in South Asia, and there is concern that this would spread to Africa, which would compromise the current ACT strategy and the use of artesunate in severe malaria [4].

Quinine has become the second-line of treatment of uncomplicated malaria in many countries [1]. However, evidence indicates that resistance to this drug is also spreading in South Asia [5]. Thus, to counterbalance this burgeoning drug resistance problem, new drugs are urgently needed.

Several investigators have demonstrated that the anti-cancer methotrexate (MTX) and trimetrexate (TMX), which are inhibitors of dihydrofolate reductase (DHFR), are potent against both pyrimethamine (PM) sensitive and PM-resistant *P. falciparum *strains (parasites harboring wild type or mutant *dhfr *respectively), including those carrying the Ileu-164-Leu *dhfr *codon, with IC_50 _< 85 nM (inhibitory concentration that kill 50% of parasitaemia) [6-10].

MTX is used at high dose, up to 5 to 24 g per adult per week (130-300 mg/kg) for several weeks for the treatment of cancer. This dose can yield serum concentrations of >1000 μM, a concentration range associated with MTX life-threatening toxicity [11]. By contrast, a 1000-fold lower dose of MTX (LD-MTX) [0.1-0.4 mg/kg (7.5-30 mg per adult)] is used once weekly in the treatment of rheumatoid arthritis (RA), juvenile idiopathic arthritis (JIA) in children (including infants <one year old) and psoriasis [12-14]. This use of LD-MTX is relatively safe and well tolerated, and children tolerate it better than adults [14]. Pharmacokinetic data indicate that LD-MTX yields *in vivo *concentrations >250 nM, which can clear malaria parasites *in vivo *(MTX IC_99 _of 200-400 nM) [15-17]. In addition, two old and small clinical trials carried out in the 1970 s, indicated that LD-MTX, as low as 2.5 mg per dose per day, was safe and efficacious to treat *P. falciparum *and *Plasmodium vivax *in adults [18,19]. Taken together, this information has led us to re-evaluate the potential of LD-MTX as anti-malarial. The literature is replete with similar examples of the use of anti-cancers at low and safe dose for the treatment of non-neoplastic diseases [20].

This is a report of a clinical trial of LD-MTX in healthy adult Kenyans to assess its safety, tolerability and pharmacokinetics, as a step towards its development for the treatment of uncomplicated *falciparum *malaria. This trial was registered at ClinTrials.gov (NCT# 00791531). As started earlier, a dose as low as 2.5 mg/day for 3 to 5 days was found to clear malaria infection, and the use of MTX in arthritis indicates that 7.5-30 mg per week was relatively safe. Thus, a dose of 5 mg/day/5 days (for a total of 25 mg per week) was chosen for investigation since it did not exceed 30 mg per treatment (the weekly dose used in arthritis), and was double the 2.5 mg/day (previously tested in malaria).

## Methods

The study was conducted between 3^rd ^March 2009 and 31^st ^August 2009 at the Kenya Medical Research Institute (KEMRI), Nairobi. After obtaining all the required ethical approvals, the study was advertised using posters and word of mouth with messages from the approved subject information sheets. The study nurses obtained informed consent from the study participants, after which recruitment into the study began. The participants underwent pre-HIV test counselling, full physical examination and a baseline blood sample was taken. They were advised to report to the study clinic the following day, ready for admission in the study ward for 5 days, if they met the eligibility criteria. The day of admission was considered as day 0 for each volunteer. Only eligible volunteers were assigned a unique study identification (ID) number, which was linked to a pre-generated random allocation to the pharmacokinetics (PK) sampling group.

This study was reviewed and approved by the KEMRI Scientific Steering Committee, KEMRI/National Ethical Review Committee (ERC), Pharmacy and Poisons Board, Kenya and the Oxford Tropical Research Ethics Committee (OXTREC number 5608). The study was conducted in compliance with ICH-GCP guidelines and the Declaration of Helsinki, 2008 [21,22].

### Study population and follow up

**Figure 1:**
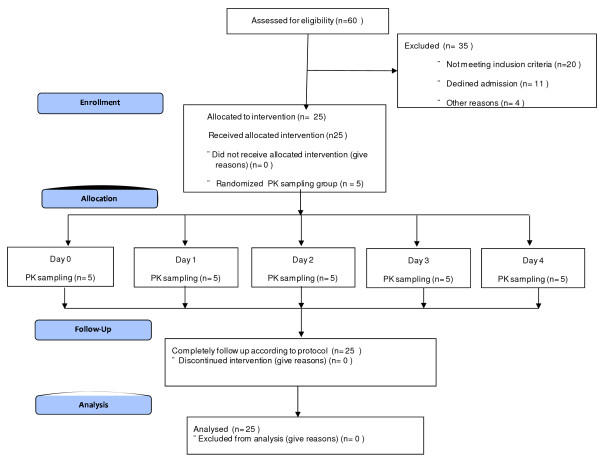
**Study flow chart**.

The study population was a group of 25 healthy adult male volunteers screened from communities living around the KEMRI facility in Nairobi. A summary of the inclusion and exclusion criteria applied to this study is shown in Table 1. Eligible volunteers were administered 5 mg of MTX daily orally under observation. All volunteers were discharged on day 5 and follow up visits were on days 7, 14, 28 and 42. Figure 1 shows the study flow chart.

**Table 1:** Summary of inclusion and exclusion criteria

Inclusion Criteria	Exclusion Criteria
Male aged ≥18 -≤55 years old; Weighing 50-75 kg Haemoglobin ≥10 g/dl HIV negative Written informed consent from the study subject.	Severe underlying conditions such as malnutrition, clinically suspected cardiac, renal, or hepatic diseases, suspected AIDS, or severe injury. Presence of any concomitant illnesses such as malaria, lower respiratory tract infections (LRTI), acute bloody or non-bloody diarrhoeas, or other acute infections. History of treatment with antimalarial drugs within the last 2 weeks. History of treatment with aspirin or any non-steroidal-anti-inflammatory agent or trimethoprim and co-trimoxazole within the last 7 days. Any ongoing medication. Abnormal clinical chemistry or hematological finding. Alcohol/drugs intake. Any other reason in the recruiting clinician's opinion that makes the individual unsuitable for taking part in a clinical trial.

### Investigational product

MTX was purchased from Wyeth Pharmaceuticals (South Africa) as 2.5 mg tablets, with a shelf-life of three years. The drug was stored in a dry, locked cabinet at a temperature below 30°C and it was used according to the manufacturer's specifications. At each dosing time point, two tablets were administered with water in the morning before breakfast every day for five days.

### Pharmacokinetics (PK), blood Sampling and measurement of MTX concentrations

The 25 study ID numbers were randomly pre-allocated (using a computer-generated simple randomization list) to five groups of five participants in each group. This allocation ensured that on each day, only one group of five participants was scheduled to give blood sample (2 ml) at 2, 4, 6, 12 and 24 hours after each daily MTX dose administration for PK study. Blood samples were collected in EDTA tubes and stored at -20°C. Samples were later transported to the KEMRI-Wellcome Trust Programme, Kilifi for analysis. Plasma concentrations of MTX were measured using Abbott TDx FLx fluorescence polarisation immunoassay analyser (Abbott Diagnostics, IL, USA). The MTX II reagent, calibrator and control kits, and diluent buffer were supplied by the manufacturer. The inter-assay coefficients of variation over the range of low (0.07 μmol/L), medium (0.4 μmol/L) and high (0.8 μmol/L) concentrations of the quality control standards were 9.23, 3.95 and 3.32% (n = 12 in all cases), respectively. Pharmacokinetic parameters calculated were maximum concentration (C_max_), half-life (T_1/2_) and area under the curve (AUC).

### Clinical safety evaluation and reporting of adverse event

Volunteers were clinically assessed before administration of each dose of study medication and on the scheduled clinic visits on days 7, 14, 28 and 42, and they were advised to return to the facility at any time when they did not feel well. The definitions for adverse events (AE); serious adverse events (SAE) and suspected unexpected serious adverse reactions (SUSARs) were used according to standard ICH-GCP [22]. SAE's, including SUSAR's were to be reported by telephone and email to the local safety monitor and sponsor within 24 hours of becoming aware of the event. The ERC and Pharmacy and Poison's Board (PPB), the regulatory authority were notified within a week.

Standardized clinical laboratory methods were employed on blood samples collected in EDTA and heparinized tubes for haematology/biochemistry and PK respectively. A, BECKMAN Coulter™ (model AC.T 5 Diff CP; Florida, USA) haematology analyzer was used with manufacturer reagents to estimate total white cell count, red cell count and haemoglobin levels. A Selectra (model E VitaLab™, The Netherlands) chemistry analyser with reagents from Randox™ (Ireland) was used to measure creatinine, alanine aminotransferase (ALT), aspertate aminotransferase (AST), alkaline phosphatase (ALP) and total bilirubin.

No formal sample size calculation was done, since this was a descriptive phase I safety study. However, because of PK considerations requiring population sampling for at least five time-points, the sample size of 25 was adopted to allow for 5 concentration-time data points for each blood sampling time-point.

### Data management and statistical methods

Because the trial involved healthy volunteers with no medical records, specific participant files were used to capture all information as source. Data was then transcribed on to case record files, which were checked by the monitors at 100% verification against the source documents. The case records where then reviewed for completeness by the data manager before entry on to the study database.

*Open*Clinica™ (an open source, GCP compliant data management system provided by Akaza Research, LLC, MA. USA), running on Microsoft Windows Server 2003, Apache Tomcat™ (an open source web server by Apache Software Foundation, California, USA) and PostgreSQL™ (open source database by PostgreSQL Global Development Group, USA) was used for setting up and managing the clinical trial data.

Standard statistical analyses, based on the review of the individual values of clinically significance variables and descriptive statistics (summary tables, graphs) were conducted using the data analysis and statistical programme, STATA (version 11); StataCorp LP, Texas, USA. Non-parametric methods were used to assess for any statistically significant changes of the clinical laboratory safety parameters.

## Results

At total of 60 adult male volunteers were screened, out of which 25 with mean age of 23.9 ± 3.3 years were enrolled in the study. Other baseline demographic characteristics collected included height, weight and body mass index (BMI), and are summarized in Table 2. A 100% protocol compliance, medication and follow-up to study procedures were achieved.

**Table 2:** Baseline demographic characteristics.

Parameters	Mean (SD)
Age (years)	23.92 (3.33)
Height (cm)	172.2 (5.14)
Weight (kg)	60.78 (4.92)
Body Mass Index (BMI)	20.5 (1.58)
Respiratory rate (breaths per min)	18.25 (0.68)
Heart rate (beats per min)	68.54 (8.57)
Systolic blood pressure (mmHg)	115.92 (7.73)
Haemoglobin level (g/dL)	16.12 (1.36)
RBC (per μL)	5.62 (0.53)
WBC (per μL)	5.69 (1.39)
ALT Test (units)	24.2 (9.58)
AST Test (units)	36.65 (13.81)
ALP Test (units)	179.35 (49.14)
Creatinine (units)	102.85 (18.83)
Total bilirubin (units	11.33 (6.20)

Among the solicited adverse events, only nausea was observed in 1/25 (4%) participant between days 3 and 7. Neither oral ulcers nor vomiting were reported at any time during the trial period. For other unsolicited symptoms, the following were reported: dizziness 2/25 (8%) and diarrhoea 1/25 (4%), and headache 3/25 (12%), and they all occurred between day 0 and 2. No other non-serious AEs were reported at follow up times till day 28 when one volunteer reported headache, diarrhoea and abdominal pains, and another volunteer complained of headache on day 42. All these events resolved without sequelae.

One serious adverse event was reported on day 21. The volunteer was brought to the study clinic and admitted in the ward in a semi-comatose state, with cough and fever. The diagnosis was right-sided lobar pneumonia with X-ray evidence of lung consolidation, which was treated with parenteral crystalline penicillin. He fully recovered and was discharged after three days without any further complications. This serious adverse event was deemed to be unrelated to the study medication.

**Figure 2:**
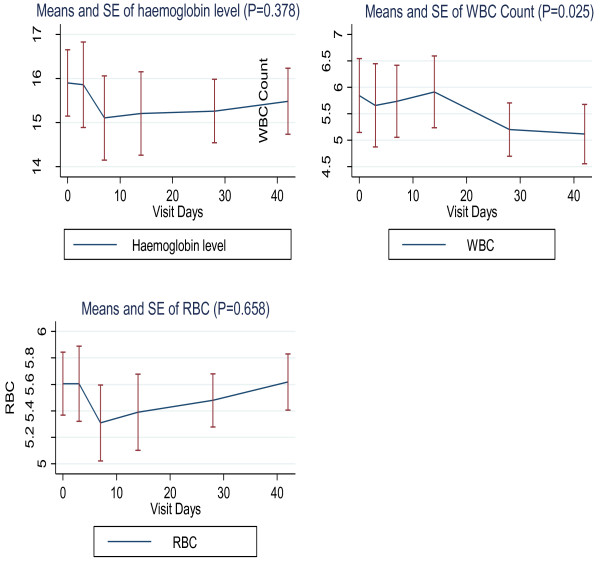
**Means and stand errors (SE) of serial haematological measurements of hemoglobin (Hb), white blood cell (WBC) and creatine (Cr)**.

For the haematological profiles, there was in general, a trend towards a mild decrease in haemoglobin, red cell count and total white blood cell count, during the first week following drug administration. However, these levels normalised by week 2-3, except those of the total white cell count, which, although remained within normal ranges, showed a statistically significant declining trend (P = 0.025). However, all these events were without any clinically significant outcome (Figure 2).

**Figure 3:**
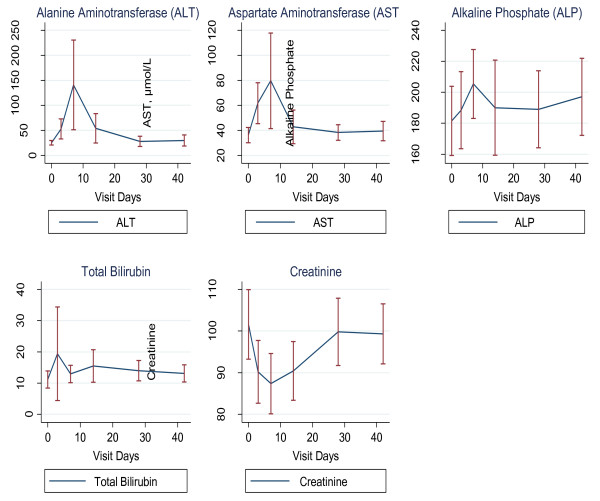
**Means and standard errors of serial clinical chemistry measurements of alanine aminotransferase (ALT), aspartate aminotransferase (AST), alkaline phosphatase (ALP) and total bilirubin (Tot. Bil)**.

Liver enzymes (AST, ALT, ALP) and total bilirubin levels tended to increase during the first week, and by the fourth week, the levels returned to normal values (Figure 3). However, one participant experienced a high (grade 3) rise in AST by up to 4.8 times and ALT 13 times higher than normal by day 7. The participant was clinically stable and asymptomatic; these abnormal parameters all returned to normal by day 14, except AST which normalized by day 28. Further investigations on any liver pathology that could potentially explain the result (including detailed medical history probing, any alcohol consumption, blood sugar and screening for hepatitis A and B) was negative.

**Figure 4:**
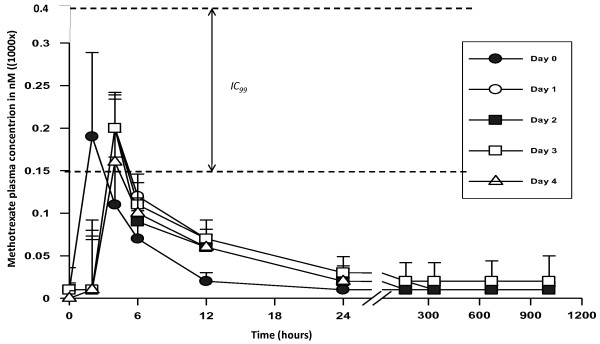
**Methotrexate (MTX) plasma concentration from day 0 to day 4**. Five participant provided blood each day, thus each point represents a mean of 5 measurements. IC_99 _represents MTX concentration that inhibits 99% of parasite growth in vitro.

PK analysis of LD-MTX (5 mg/day for 5 days) showed that C_max _ranged from 160-200 nM, and these levels were achieved within the first hour. Thereafter, concentrations rapidly declined and the effective concentration (C_eff_) was <150 nM after 6 hours, following drug administration (Figure 4). These levels are lower than the reported IC_99_, which is around 200-400 nM. The concentrations were consistently less than 50 nM by 24 hours post dose (Figure 4). The geometric means for C_max _and area under the curve from 0 to 24 H (AUC_0-24 H_) on day 0 were 170 and 959 nmoles hours/L, respectively, compared with 145 and 936 nmoles hours/L, respectively, on day 4. These results suggest that MTX does not accumulate in the body following repeated administration of the LD-MTX over 5 days. Drug levels for each participant are given in Additional file 1.

## Discussion

LD-MTX, at doses between 7.5-30 mg/adult per week for several years has been extensively used in the western world for the treatment inflammatory diseases including RA, JIA and psoriasis [12-14]. At this dose and regime, the drug is safe and well tolerated. These data confirm the safety profile of LD-MTX at 5 mg/day/5 days in an African population.

The use of MTX is known to mildly increase liver enzymes, and the levels always normalise within a few weeks after drug discontinuation [23,24]. Thus, the increase in liver enzyme levels we observed was expected, and they were all normalised within four weeks, as the data show. One participant however experienced excessively high AST and ALT enzyme levels. In the treatment of inflammatory diseases, such high rises in liver enzymes are rare, and in most cases, they are the result of the pre-existing liver infections such as hepatitis A, B, C, D, E, G, Adenoviruses, Epstein-Barr-virus, among others; alcohol use, obesity and diabetes can also contribute to a higher rise of liver enzymes [23,24]. Although all possible causes were not investigated, the following could be excluded: alcohol use, obesity, diabetes and hepatotropic virus infections, such as hepatitis A and B. Nevertheless, at day 28, the key study time point, the levels in the volunteer had normalized, and he never experienced any clinical symptoms. Overall, a significant mild decrease in white cell was observed up to day 42. It is well known that the use of LD-MTX is associated with decrease in haematological parameters (haemogloglin, red blood cells and white cells), however the levels normalized within few weeks after the end of treatment [12,13]. In the current study, white cell count were significantly decreased, but the levels were still within the normal reference ranges, and had no clinical significance. Nevertheless, this parameter should be closely monitored in all subsequent clinical evaluations of this drug.

LD-MTX is increasingly being used in Africa [25], including in Kenya [26] for the treatment of RA, JIA and psoriasis. In these situations, liver enzymes are monitored regularly as part of the management of LD-MTX treatment. In Kenya, more than 100 patients are or had been under LD-MTX treatment for several months, and no such high levels of liver enzymes has been reported (G.O. Oyoo, unpublished). Thus, it is clear that enzyme levels should be monitored closely in all our subsequent clinical evaluation of LD-MTX.

LD-MTX is also being used in the treatment of various disease conditions including: inflammatory bowel disease [27]; urticaria ([28] ankylosing spondylitis [29]; idiopathic hypertrophic cranial pachymeningitis [30]; chronic cholestatic disorder ([31]; Wegener's granulomatosis [32]; primary biliary cirrhosis [33]; systemic lupus erythematosus [34]; and inflammatory eye disease [35], haemophagocytic lymphohistiocytosis, a disease that affects younger children, including infants (<12 months of age) [36]. Worldwide, it is estimated that 0.5-1 million adults and 50,000-100,000 children receive LD-MTX weekly for the treatment of RA, JIA respectively. The most commonly reported LD-MTX side effects are nausea, vomiting, diarrhoea and oral ulcers. These side effects were solicited in this trial, and as the observations indicate, they were not commonly found at the 5 mg/day dose in the study cohort. This was expected indeed as it is well known that LD-MTX adverse effects are the result of the chronic use of the drug, and these symptoms are generally observed after several weeks of therapy. LD-MTX in this study was used for 5 days only, thus it is not surprising that these adverse effects were rare. In RA, JIA and psoriasis, folate derivatives are administered in some patients to lower these effects. However, the five-day drug regimen did not warrant use of folate derivatives. Thus, for treatment of uncomplicated malaria, LD-MTX could indeed be used alone as we proposed.

In previous work, MTX IC_50 _around 40-85 nM were reported these values correspond to IC_99 _around 200-400 nM [6,37], thus C_eff _of MTX >400 nM should achieved to clear malaria infection. However our PK analysis shows that the achieved mean C_max _was around 200 nM only, and after 6 hours, MTX concentrations declined below 150 nM. These concentrations are not high enough to clear malaria infection; concentrations above 400 nM for 6-12 hours would need to be achieved. This implies that further studies should be carried out to evaluate the dose range that will yield adequate plasma concentrations and still be well tolerated. One of the approaches would be to reduce the days of treatment from 5 to 3 only, and increase the dose to 7.5, 10 or 12.5 mg per day. The total dose for each treatment course should however remain around 22.5-37 mg, which is close to the range of doses administered weekly in the treatment of inflammatory diseases (7.5-35 mg per adult). At such doses, it is expected that, the drug would remain safe since its toxicity is mainly the result of chronic use, while in the case of malaria treatment, it would be used for three days only. Another approach could be to start with a loading dose of 10 to 15 mg for the first day, and subsequently use lower doses to 5 or 7.5 mg, a strategy similarly used with quinine and artemether/lumefantrine in the treatment of severe and uncomplicated malaria, respectively [1].

In conclusion, 5 mg of MTX/day for 5 days is safe and well tolerated. However, MTX blood levels achieved are not high enough to clear malaria infection. Therefore, other dose ranging clinical investigation need to be carried out to establish the optimal doses that are safe and can yield adequate MTX concentrations in the body.

## Competing interests

The authors declare that they have no competing interests.

## Authors' contributions

RC, AA, MB, RJ, TL, AN, OO, TN and KM were involved in the conception of the project, study design, protocol development, and overall organization of the study. HL, AA, HL, PN, GM, AS, EA were involved in the implementation of trial, data collection, coordination, and monitoring of the study. EW was the manager who created the database and managed data. RC, JJ, AN, TL, HL, CK and MB were involved in data analysis and interpretation of results. RC and AA developed and coordinated manuscript writing. GK and SNM contributed in the study design, drug level measurement and pharmacokinetic data analysis.

All the authors read and approved the final manuscript.

## Financial support

This study was supported by the European Developing Countries Clinical Trials Partnership (EDCTP) and the Wellcome Trust WT077092 (to Prof Kevin Marsh for activity of the KEMRI/Wellcome Trust programme).

## Supplementary Material

Additional file 1 **Mean and standard deviation (SD) of plasma methotrexate concentrations (1000 × nmol/L)**. As explained in Material and Methods, the 25 participants were randomly selected in group of five, for a total of 5 groups. Each day, only one group was scheduled to give blood sample after each daily MTX dose administration for pharmacokinetic analysis (PK). Tables A, B, C, D, E represent data for participants selected for day 0 (the first day of the study), day1, day 2, day 3, and day 4 respectively. All participants were followed up on 7, 14, 28 and 42 days, and blooded collected for PK analysis on each of these aforementioned day. ND stands for "not determined". Click here for file
