# Supplementary material for: A phase I trial to evaluate the safety and pharmacokinetics of low-dose methotrexate as an anti-malarial drug in Kenyan adult healthy volunteers
Source: Malar J. 2011 Mar 16;10:63. doi: 10.1186/1475-2875-10-63 (PMC3072355; doi:10.1186/1475-2875-10-63)
Supplement: Additional file 1 — Mean and standard deviation (SD) of plasma methotrexate concentrations (1000 × nmol/L). As explained in Material and Methods, the 25 participants were randomly selected in group of five, for a total of 5 groups. Each day, only one group was scheduled to give blood sample after each daily MTX dose administration for pharmacokinetic analysis (PK). Tables A, B, C, D, E represent data for participants selected for day 0 (the first day of the study), day1, day 2, day 3, and day 4 respectively. All participants were followed up on 7, 14, 28 and 42 days, and blooded collected for PK analysis on each of these aforementioned day. ND stands for "not determined". [file 1475-2875-10-63-S1.DOC]

**Additional file 1 -** Mean and standard deviation (SD) of plasma methotrexate concentrations (1000x nmol/L).

**A.**

| **Sample**  **Time (hours)** | **Sample**  **day** | **Study subject identification number** | | | | | **Mean+SD** |
| --- | --- | --- | --- | --- | --- | --- | --- |
|  |  | **A01** | **A06** | **A11** | **A16** | **A21** |  |
| 0 | Day 0 | 0.00 | 0.02 | 0.01 | 0.01 | 0.00 | 0.01+0.008 |
| 2 | Day 0 | 0.32 | 0.11 | 0.25 | 0.08 | 0.20 | 0.19+0.099 |
| 4 | Day 0 | 0.18 | 0.06 | 0.11 | 0.05 | 0.15 | 0.11+0.056 |
| 6 | Day 0 | 0.10 | 0.04 | 0.07 | 0.03 | 0.10 | 0.07+0.033 |
| 12 | Day 0 | 0.03 | 0.01 | 0.02 | 0.01 | 0.03 | 0.02+0.010 |
| 24 | Day 0 | ND | 0.00 | 0.02 | 0.01 | 0.02 | 0.01+0.010 |
| 168 | Day 7 | ND | 0.01 | ND | 0.01 | 0.00 | 0.01+0.006 |
| 336 | Day 14 | 0.01 | 0.01 | 0.01 | 0.02 | 0.02 | 0.01+0.005 |
| 672 | Day 28 | 0.01 | 0.01 | 0.01 | 0.01 | 0.01 | 0.01+0.000 |
| 1008 | Day 42 | 0.01 | 0.01 | 0.00 | 0.01 | 0.01 | 0.01+0.004 |

**B**

| **Sample**  **time** | **Sample**  **day** | **Study subject identification number** | | | | | **Mean** |
| --- | --- | --- | --- | --- | --- | --- | --- |
|  |  | **B02** | **B07** | **B12** | **B17** | **B22** |  |
| Pre**-**dose | DAY 0 | ND | 0.01 | 0.00 | 0.01 | 0.01 | 0.01+0.005 |
| 0 | DAY 1 | ND | 0.01 | 0.01 | 0.00 | 0.00 | 0.01+0.006 |
| 2 | DAY 1 | 0.15 | 0.20 | 0.31 | 0.17 | 0.18 | 0.20+0.063 |
| 4 | DAY 1 | 0.10 | 0.11 | 0.19 | 0.13 | 0.08 | 0.12+0.042 |
| 6 | DAY 1 | 0.06 | 0.07 | 0.11 | 0.08 | 0.04 | 0.07+0.026 |
| 12 | DAY 1 | 0.01 | 0.03 | 0.03 | 0.04 | 0.03 | 0.03+0.011 |
| 24 | DAY 1 | ND | 0.02 | 0.02 | 0.02 | 0.01 | 0.02+0.005 |
| 168 | DAY 7 | ND | 0.02 | 0.01 | 0.01 | 0.01 | 0.01+0.005 |
| 336 | DAY 14 | ND | 0.01 | 0.01 | 0.01 | 0.01 | 0.01+0.000 |
| 672 | DAY 28 | ND | 0.02 | 0.01 | 0.01 | 0.01 | 0.01+0.005 |
| 1008 | DAY 42 | ND | ND | ND | ND | ND | ND |

**C.**

| **Sample**  **time** | **Sample**  **day** | **Study subject identification number** | | | | | **Mean** |
| --- | --- | --- | --- | --- | --- | --- | --- |
|  |  | **D04** | **D09** | **D14** | **D19** | **D24** |  |
| Predose | DAY 0 | 0.00 | 0.01 | 0.01 | 0.00 | 0.01 | 0.01+0.005 |
| 0 | DAY 2 | 0.00 | 0.01 | 0.01 | 0.00 | 0.02 | 0.01+0.008 |
| 2 | DAY 2 | 0.21 | 0.31 | 0.24 | 0.11 | 0.13 | 0.20+0.082 |
| 4 | DAY 2 | 0.07 | 0.14 | 0.12 | 0.07 | 0.07 | 0.09+ 0.034 |
| 6 | DAY 2 | 0.04 | 0.09 | 0.07 | 0.04 | 0.04 | 0.06+0.023 |
| 12 | DAY 2 | 0.00 | 0.02 | 0.04 | 0.02 | 0.02 | 0.02+0.014 |
| 24 | DAY 2 | 0.00 | 0.04 | 0.02 | 0.00 | 0.00 | 0.01+0.018 |
| 168 | DAY 7 | 0.00 | 0.02 | 0.01 | 0.00 | 0.02 | 0.01+0.010 |
| 336 | DAY 14 | 0.00 | 0.01 | 0.02 | 0.00 | 0.01 | 0.01+0.008 |
| 672 | DAY 28 | 0.00 | 0.01 | 0.01 | 0.00 | 0.01 | 0.01+0.005 |
| 1008 | DAY 42 | ND | ND | ND | ND | ND | ND |

**D.**

| **Sample**  **time** | **Sample**  **day** | **Study subject identification number** | | | | | **Mean** |
| --- | --- | --- | --- | --- | --- | --- | --- |
|  |  | **C03** | **C08** | **C13** | **C18** | **C23** |  |
| Predose | DAY 0 | 0.00 | 0.01 | 0.00 | 0.05 | 0.01 | 0.01+0.021 |
| 0 | DAY 3 | 0.00 | 0.01 | 0.00 | 0.06 | 0.00 | 0.01+0.026 |
| 2 | DAY 3 | 0.24 | 0.10 | 0.19 | 0.25 | 0.20 | 0.20+0.059 |
| 4 | DAY 3 | 0.11 | 0.07 | 0.08 | 0.17 | 0.11 | 0.11+0.039 |
| 6 | DAY 3 | 0.06 | 0.04 | 0.06 | 0.11 | 0.07 | 0.07+0.026 |
| 12 | DAY 3 | 0.02 | 0.02 | 0.02 | 0.07 | 0.02 | 0.03+0.022 |
| 24 | DAY 3 | 0.00 | 0.01 | 0.01 | 0.05 | 0.01 | 0.02+0.019 |
| 168 | DAY 7 | ND | 0.01 | 0.00 | 0.05 | 0.01 | 0.02+0.022 |
| 336 | DAY 14 | ND | 0.01 | 0.00 | 0.05 | 0.01 | 0.02+0.022 |
| 672 | DAY 28 | ND | 0.01 | 0.00 | 0.05 | 0.00 | 0.02+0.024 |
| 1008 | DAY 42 | ND | ND | ND | ND | ND | ND |

**E.**

| **Sample**  **time** | **Sample**  **day** | **Study subject identification number** | | | | | **Mean** |
| --- | --- | --- | --- | --- | --- | --- | --- |
|  |  | **E05** | **E10** | **E15** | **E20** | **E25** |  |
| Predose | DAY 0 | 0.01 | 0.00 | 0.01 | 0.00 | 0.00 | 0.00+0.005 |
| 0 | DAY 4 | 0.02 | 0.01 | 0.00 | 0.00 | 0.00 | 0.01+0.009 |
| 2 | DAY 4 | 0.23 | 0.09 | 0.15 | 0.09 | 0.23 | 0.16+0.070 |
| 4 | DAY 4 | 0.13 | 0.06 | 0.10 | 0.07 | 0.14 | 0.10+0.035 |
| 6 | DAY 4 | 0.08 | 0.04 | 0.05 | 0.04 | 0.07 | 0.06+0.018 |
| 12 | DAY 4 | 0.02 | 0.02 | 0.03 | 0.02 | 0.02 | 0.02+0.004 |
| 24 | DAY 4 | 0.01 | 0.01 | 0.01 | 0.01 | 0.00 | 0.01+0.00 |
| 168 | DAY 7 | 0.01 | 0.00 | ND | 0.01 | 0.00 | 0.01+0.006 |
| 336 | DAY 14 | 0.01 | 0.00 | 0.01 | 0.01 | 0.00 | 0.01+0.005 |
| 672 | DAY 28 | 0.02 | 0.01 | 0.01 | 0.02 | 0.00 | 0.01+0.008 |
| 1008 | DAY 42 | ND | ND | ND | ND | ND | ND |
